# Ferroptosis: an emerging key mechanism linking aging, surgical and anesthetic exposure to postoperative cognitive dysfunction

**DOI:** 10.3389/fimmu.2026.1836516

**Published:** 2026-05-22

**Authors:** Danxia Jiang, Qiaolan Zhang, Yin Tong, Kecheng Lou, Haicheng Liu

**Affiliations:** 1 The Second Clinical Medical College of Zhejiang Chinese Medical University, Hangzhou, China; 2 Department of Urology, The Sixth People's Hospital of Huizhou, Huizhou, Guangdong, China; 3 Department of Urology, Lanxi People's Hospital, Jinhua, Zhejiang, China

**Keywords:** aging, ferroptosis, iron metabolism, neuroinflammation, postoperative cognitive dysfunction

## Abstract

Postoperative cognitive dysfunction (POCD) is a common complication in older surgical patients. While its pathogenesis remains unclear, ferroptosis—an iron-dependent form of cell death driven by lipid peroxidation—has emerged as a key mechanism in neurodegeneration. This review proposes that aging creates a ferroptosis-prone environment in the brain through iron dyshomeostasis, impaired antioxidant defenses, and enrichment of polyunsaturated fatty acids, and that surgical trauma and anesthetic exposure may trigger ferroptosis by activating interconnected pathways such as neuroinflammation, blood-brain barrier disruption, and oxidative stress, leading to neuronal injury in cognition-critical regions like the hippocampus. However, the available evidence is largely correlative, and whether ferroptosis acts as a proximal driver of neuronal death or as a late consequence of pre-existing damage remains undetermined. We dissect the core molecular machinery (GPX4, ACSL4, NCOA4, Nrf2) and emerging regulators (MD2/Hepcidin, CPT1A, RUNX1/RBM47/cGAS-STING, miRNAs, mitophagy, gut microbiota-exosome axis). Therapeutic strategies including iron chelators, lipophilic antioxidants, natural products, physical therapies, and nanomaterials are reviewed, but most remain preclinical. Elucidating the role of ferroptosis may open new avenues for early diagnosis, targeted prevention, and effective treatment, provided that causality can be rigorously established.

## 1 Introduction

Postoperative cognitive dysfunction (POCD) is a common complication affecting the central nervous system following surgery and anesthesia, with a particularly high incidence in elderly patients. It severely impacts postoperative recovery, quality of life, and even long-term survival rates. Statistically, approximately 10%-54% of patients experience POCD in the initial weeks following surgery (1), and cognitive deficits persist at 3 months post-surgery in 12%-17% of patients (2). The clinical manifestations of POCD are diverse, with core features encompassing declines in memory, attention, executive function, information processing speed, and verbal fluency (3). The underlying mechanisms are complex and not fully elucidated, but current research generally implicates neuroinflammation, oxidative stress, neurotransmitter imbalance, mitochondrial dysfunction, and altered synaptic plasticity as key pathological processes (4). Furthermore, patient age, preoperative cognitive reserve, type of surgery, anesthetic technique, postoperative pain management, and perioperative complications significantly influence the development and progression of POCD (5, 6). Against the backdrop of a globally aging population, the high incidence of POCD not only prolongs hospital stays (7) and increases healthcare burdens but may also trigger or accelerate other neurodegenerative diseases, imposing substantial burdens on patients, families, and society.

Current research on POCD mechanisms predominantly focuses on neuroinflammation and neurotransmitter systems. The prevailing hypothesis suggests that surgical trauma can induce a systemic inflammatory “storm.” These peripheral inflammatory mediators can cross the compromised or more permeable blood-brain barrier (BBB) to access the central nervous system, activating microglia and triggering an inflammatory cascade within the brain (8). Anesthetic drugs may also directly interfere with neuronal enzyme activities, affect neurotransmitter synthesis and release, and disrupt synaptic information transmission, thereby reducing neural network stability. However, the initiating factors, core drivers, and the intricate interplay among these pathological processes (such as neuroinflammation, mitochondrial dysfunction (9), and protein homeostasis disruption) in POCD remain unclear. Consequently, accurately predicting and identifying high-risk individuals for POCD in clinical practice is challenging, and interventions based on anti-inflammatory strategies or inhibition of microglial activation have not yielded satisfactory results in clinical translation (10). Unlike Alzheimer’s disease (AD), which is characterized by specific pathological protein deposits (e.g., Aβ and Tau) (11), POCD lacks specific biomarkers and standardized pharmacological treatments, revealing significant shortcomings in current clinical prevention and management strategies. This dilemma suggests that a more upstream, core pathological mechanism, capable of integrating the disparate elements of aging, surgical trauma, anesthetic exposure, and cognitive decline, may have been overlooked. We propose that this core mechanism is ferroptosis.

Ferroptosis, a term first coined by Dixon et al. in 2012, is a form of regulated cell death characterized by the iron-dependent lethal accumulation of lipid peroxides (12). Morphologically and biochemically, ferroptosis differs markedly from other forms of cell death, such as apoptosis, necrosis, and autophagy: morphologically, it is characterized by an intact cell membrane, mitochondrial shrinkage, and a reduction or disappearance of cristae, while the nucleus shows no obvious chromatin condensation or marginalization (13); biochemically, it is primarily associated with depletion of intracellular glutathione (GSH) and inactivation of glutathione peroxidase 4 (GPX4), leading to impaired clearance and accumulation of membrane lipid peroxides (14). In recent years, a substantial body of research has confirmed a close association between abnormal iron deposition in specific brain regions and the pathological progression of various neurodegenerative diseases, including Parkinson’s disease (PD), Alzheimer’s disease (AD), amyotrophic lateral sclerosis (ALS), and Huntington’s disease (HD) (15). This strongly suggests that ferroptosis may play a critical role in neuronal loss and cognitive decline, offering a novel perspective for understanding the pathological essence of POCD.

Elderly patients represent the highest risk group for POCD, with an incidence significantly exceeding that in younger populations. This points to the unique physiological microenvironment of the “aged brain” as the basis for POCD susceptibility (16). Notably, iron dyshomeostasis and a propensity for ferroptosis are themselves core features of aging. With advancing age, although circulating iron levels may decrease, storage iron within tissues and cells paradoxically increases. This redistribution of iron, coupled with reciprocal promotion of redox imbalance, facilitates ferroptosis and accelerates the aging process (17). Simultaneously, brain tissue is rich in polyunsaturated fatty acids (PUFAs), which are highly susceptible to lipid peroxidation. Ferroptosis resulting from abnormal lipid metabolism can exacerbate acute central nervous system injury and create a vicious cycle of iron overload in specific brain regions (18). Upon this susceptible substrate, perioperative stressors like surgical trauma and anesthesia act as catalysts. They further promote abnormal iron accumulation and distribution within the brain by increasing BBB permeability (19), facilitating the massive release of peripheral pro-inflammatory cytokines (20), and exacerbating oxidative stress (21). Excess iron catalyzes the production of highly reactive hydroxyl radicals via the Fenton reaction, which vigorously attacks neuronal membranes rich in PUFAs, initiating a chain reaction of lipid peroxidation. This ultimately triggers ferroptosis in neurons within critical cognitive regions like the hippocampus (22), leading to synaptic dysfunction, extensive neuronal damage, and the manifestation of POCD.

Based on this, we propose a testable hypothesis in which aging constitutes the permissive “soil,” surgery and anesthesia act as “catalysts,” and ferroptosis serves as a central integrating mechanism ultimately leading to cognitive impairment. However, as detailed below, most supporting evidence is derived from animal models and correlative biomarker studies; ferroptosis may also represent a downstream consequence of neuroinflammation and oxidative stress rather than its primary driver. This review aims to critically examine the evidence for ferroptosis as a causal mechanism in POCD, to identify knowledge gaps, and to outline the experimental and clinical steps needed to validate this framework.

## 2 Ferroptosis susceptibility in the aged brain: the predisposing basis for POCD development

Aging is the most significant independent risk factor for POCD, arising from a “ferroptosis-prone state” established in the aged central nervous system due to alterations in iron metabolism, antioxidant defenses, and lipid composition.

Iron dyshomeostasis is a hallmark of aging and is closely linked to the pathogenesis of neurodegenerative diseases like AD and PD. Masaldan et al. demonstrated significant iron accumulation in aging mouse fibroblasts, associated with impaired ferritinophagy (23), suggesting that senescent cells inherently exist in a pro-ferroptotic state. Consequently, it has been proposed that brain cell senescence in conditions like AD might be promoted by iron overload (24). Furthermore, elevated iron load in the aging hippocampus has been linked to AD symptomatology (25). In AD patients and transgenic mouse models, excess iron deposits within insoluble Aβ plaques and neurofibrillary tangles (26–28). Ferroportin 1 (Fpn), the only known mammalian non-heme iron exporter, plays a crucial role in maintaining iron homeostasis (29). However, downregulation of Fpn expression has been observed in the brains of AD patients and APPswe/PS1dE9 double transgenic mice (30–32), directly leading to impaired iron efflux and intracellular retention. Advanced imaging techniques have confirmed the association between iron deposition and core AD pathology *in vivo*. A 2020 study combining quantitative susceptibility mapping (QSM) and tau-PET found a positive correlation between iron deposition in the inferior temporal gyrus and insoluble tau aggregates in AD patients (33). Using novel fluorescent turn-on sensors, Wu et al. observed not only elevated iron levels associated with Aβ plaque deposition but, critically, an increased ratio of ferric iron (Fe³^+^) to ferrous iron (Fe²^+^) in the cortex of AD mouse models (34), directly indicating an increase in substrates for the Fenton reaction and the occurrence of ferroptosis.

The blood-brain barrier (BBB) serves as the “gatekeeper” of the central nervous system, providing a highly stable microenvironment for neuronal activity (35). Its core structure, the cerebral microvascular endothelial cells, tightly regulates brain iron homeostasis through specialized transporters: transferrin-bound iron (Tf-Fe) is internalized via transferrin receptor 1 (TfR1), non-transferrin-bound iron (NTBI) is primarily taken up by divalent metal transporter 1 (DMT1), and the crucial pathway for iron release into the brain is the sole exporter Fpn. Additionally, H-ferritin itself can be transported as an iron source (36). This precise regulation deteriorates during aging. Dynamic contrast-enhanced magnetic resonance imaging (DCE-MRI) quantifying BBB permeability in the living human brain has revealed an age-dependent decline in BBB integrity in the hippocampus, particularly in the CA1 and dentate gyrus subregions, showing significantly increased permeability (37). This allows more peripheral iron, including serum ferritin which increases with age, to enter the brain parenchyma. Interestingly, one study found that in men aged 65 and older, higher serum ferritin levels were paradoxically associated with better executive and language functions (36), potentially representing compensatory homeostatic regulation dependent on hepatic hepcidin’s fine-tuning of systemic iron metabolism (38). However, when aging or chronic inflammation disrupts hepatic hepcidin signaling, concurrent with increased hippocampal BBB permeability, this homeostasis is broken. This leads to aberrant iron cycling and excessive H-ferritin influx into the brain, ultimately culminating in brain iron overload, progressive hippocampal-cortical atrophy, and neuronal loss (39).

The generation of reactive oxygen species (ROS) and reactive nitrogen species (RNS) increases with age (40), positioning aging itself as a state of chronic oxidative stress, accompanied by declining mitochondrial oxidative phosphorylation efficiency. Research on brain aging confirms that alongside progressive iron accumulation, there is a concurrent decline in the expression and activity of key antioxidant components like GSH and GPX4 (41). The combination of these factors renders the aged brain in a state of deficient antioxidant defense but sufficient pro-oxidant substrate (iron), a “sub-ferroptotic state.” Excess ROS attacks cellular components. Zhou et al. found that in a sevoflurane-induced cognitive impairment model in aged mice, ROS generation mediated microglial pyroptosis via NLRP3 inflammasome activation. The ROS scavenger N-acetylcysteine (NAC) not only mitigated morphological changes in cells but also significantly improved cognitive function in aged mice (42), highlighting the core role of oxidative stress in anesthetic neurotoxicity.

Brain tissue, particularly highly specialized neurons, is enriched with PUFAs. The double bonds in their molecular structure render the hydrogen atoms on methylene groups highly susceptible to abstraction by ROS, initiating and propagating the chain reaction of lipid peroxidation (43). This constitutes another intrinsic reason for the brain’s vulnerability to ferroptosis. Aging-related iron accumulation exhibits regional specificity, primarily affecting the basal ganglia and other brain regions associated with motor function (44), explaining the higher incidence of movement disorders like PD in the elderly. Notably, glial cells contain significantly higher iron concentrations than neurons. Studies have found that iron concentration in oligodendrocytes of the neocortex is five times higher than in neurons, with microglia being three times and astrocytes twice as high (45). Astrocytes, as the primary regulators of CNS iron homeostasis, undergo crucial functional changes during aging. Comparative studies in mice of different ages revealed increased concentrations of iron, copper, and zinc in the basal ganglia with age, accompanied by a simultaneous increase in the numbers of microglia and astrocytes (46). In the aging human brain, increased iron staining intensity is also primarily observed in astrocytes and microglia. This suggests that age-related astrogliosis and microgliosis, along with functional alterations, may be key upstream events leading to cerebral iron dyshomeostasis and iron overload in the neuronal microenvironment.

In summary, the unique physiological environment of the aged brain—characterized by fragile iron homeostasis, diminished antioxidant capacity, and an abundance of peroxidation-prone PUFAs—constitutes a permissive environment for ferroptosis. This is the fundamental reason for the high incidence of POCD in the elderly population.

## 3 The triggering role of surgery and anesthesia in POCD development

Building upon the susceptible foundation laid by aging, surgery and anesthesia, as significant perioperative stressors, act as catalysts that rapidly amplify and ultimately trigger ferroptosis in the brain through multiple interconnected molecular pathways.

### 3.1 Neurotoxic mechanisms of the inhalational anesthetic sevoflurane

Sevoflurane, an inhalational anesthetic widely used for its safety and efficacy, has also been implicated in potentially promoting POCD (47). A substantial body of research indicates that sevoflurane can directly interfere with neuronal iron metabolism. It decreases the expression of iron regulatory protein 2 (IRP2) and the iron importer TfR1, while increasing expression of the iron storage protein ferritin in cultured rat hippocampal neurons. This altered regulatory pattern leads to intracellular iron overload (47, 48). Sevoflurane also causes significant mitochondrial damage by inhibiting mitofusin 2 (Mfn2), increasing mitochondria-endoplasmic reticulum contact, and inducing mitochondrial calcium overload and fragmentation. This results in increased opening of the mitochondrial permeability transition pore (mPTP), decreased mitochondrial membrane potential (MMP), and reduced ATP production (48). Importantly, the iron chelator deferiprone (DFP) effectively reverses these sevoflurane-induced neurotoxic effects (47, 48), directly demonstrating the critical role of iron overload. At the level of anesthetic targets, sevoflurane acts partly through N-methyl-d-aspartate (NMDA) receptor excitation. NMDA receptor activation itself can enhance DMT1-mediated iron influx via a signaling cascade involving Dexras1 (49) and stimulate lysosomal iron release (50), thereby exacerbating iron overload.

Research by Gu et al. further elucidated upstream molecular events in sevoflurane-triggered ferroptosis. They found that sevoflurane exposure activates the JNK/p38 MAPK pathway in the neonatal mouse brain, leading to intracellular ROS accumulation and DNA damage. ATM/p53, as core sensors of DNA damage, are activated. This activation upregulates TfR1 and downregulates Fpn, increasing intracellular ferrous iron, while simultaneously promoting lipid peroxidation through enzymes like NOX4, ALOX12, and ALOX5. Thus, ATM/p53 activation drives neuronal ferroptosis via both “iron supply” and “fire production” mechanisms. *In vivo* experiments confirmed that repeated sevoflurane exposure not only caused pyramidal neuron death in the hippocampal CA1 region of neonatal mice but also resulted in persistent deficits in spatial learning and memory. Iron chelators, JNK/p38 inhibitors, ROS scavengers, or p53 inhibitors all alleviated these impairments to varying degrees (51). Furthermore, from an epigenetic perspective, Li et al. discovered that myricetin reverses sevoflurane-induced upregulation of histone deacetylase 2 (HDAC2) and deacetylation of Nrf2, thereby activating the Nrf2/HO-1 signaling pathway. This inhibits hippocampal neuronal ferroptosis and mitochondrial dysfunction, ultimately alleviating cognitive impairment in aged mice (52). This suggests that sevoflurane may suppress the body’s antioxidant defenses by influencing protein acetylation modifications.

Further research has identified novel targets regulating sevoflurane neurotoxicity. Cheng et al. found that sevoflurane induces ferroptosis in SH-SY5Y cells, an effect mediated partly through upregulation of acyl-CoA synthetase long-chain family member 4 (ACSL4). Silencing ACSL4 protected cells from sevoflurane-induced ferroptosis by activating the AMPK/mTOR signaling pathway, increasing cell viability and GPX4 levels, while decreasing iron, ROS, and lipid peroxide levels (53). This identifies ACSL4 as a key executor of sevoflurane’s neurotoxicity. Another study revealed a novel role for the E3 ubiquitin ligase Mind bomb-2 (MIB2). Sevoflurane anesthesia increased MIB2 expression in the hippocampus of aged mice. MIB2 directly bound to GPX4 and promoted its ubiquitination and degradation, reducing GPX4 stability and thereby weakening the cellular defense against ferroptosis. Knocking down MIB2 attenuated sevoflurane-induced neuronal death, ferroptosis, and cognitive impairment (54). Additionally, bioinformatics analysis based on single-cell RNA sequencing identified significant upregulation of PLCG1 in the mouse hippocampus following sevoflurane exposure, primarily localized in astrocytes and neurons. Elevated PLCG1 enhanced mitochondrial oxidative stress and promoted neuronal ferroptosis by facilitating the ubiquitination of LAMP2A, thereby inhibiting chaperone-mediated autophagy (55). These studies collectively depict a complex network through which sevoflurane synergistically induces ferroptosis via multiple targets and pathways. Recent research has also implicated microRNA-135b-5p (miR-135b-5p) in sevoflurane-induced iron dysregulation by targeting the JAK2-STAT3 pathway to modulate hepcidin expression. In a mouse model of sevoflurane exposure, overexpression of miR-135b-5p inhibited JAK2-STAT3 activation, reduced hepcidin, restored Fpn1 expression, and consequently alleviated iron overload and cognitive impairment (56). This highlights the role of non-coding RNAs in the epigenetic regulation of anesthetic neurotoxicity.

### 3.2 Dual effects of intravenous anesthetics

Intravenous anesthetics primarily exert their effects by enhancing γ-aminobutyric acid (GABA) receptor function (47). Similar to sevoflurane, some intravenous agents like etomidate have been reported to induce ferroptosis and mitochondrial damage by generating excessive ROS and causing cellular iron overload. However, interestingly, propofol, the most commonly used clinical intravenous anesthetic, possesses intrinsic antioxidant and lipid peroxidation inhibitory capabilities due to its structural similarity to the antioxidant vitamin E (57), demonstrating potential neuroprotective effects.

Fan et al. confirmed this protective effect of propofol. In a mouse model of cerebral ischemia-reperfusion, propofol preconditioning significantly inhibited neuronal ferroptosis by activating the Nrf2/GPX4 signaling pathway, alleviating pathological damage, and improving motor and sensory function (58). In a traumatic brain injury (TBI) model, Zheng et al. found that propofol improved cognitive function in mice by modulating the endothelial nitric oxide synthase (eNOS)/NO signaling pathway. This elevated post-injury reduced GSH levels, decreased malondialdehyde (MDA) and ROS, and inhibited neuronal apoptosis, astrogliosis, and microglial activation (59). These studies suggest propofol possesses multiple neuroprotective mechanisms. Further advancing this concept, a study comparing propofol and isoflurane in aged rats undergoing splenectomy found that the propofol anesthesia group, compared to the isoflurane group, exhibited significantly less cognitive impairment. This was accompanied by inhibition of apoptosis, oxidative stress, neuroinflammation, iron deposition, and ferroptosis markers (e.g., upregulation of ACSL4, TfR1; downregulation of SLC7A11, FTH1) in the hippocampal CA1 region. Molecular docking suggested propofol could bind with high affinity to SIRT1, exerting its protective effects by activating the SIRT1/Nrf2/GPX4 signaling pathway, an effect reversed by a SIRT1 inhibitor (60). This provides a solid molecular basis for the cognitive protective advantage of propofol over inhalational anesthetics.

However, these neuroprotective effects demonstrated in preclinical models must be weighed against potential clinical hazards. Propofol infusion syndrome, a rare but life-threatening condition characterized by mitochondrial dysfunction, metabolic acidosis, and rhabdomyolysis, has been reported especially with high-dose prolonged infusion. Moreover, propofol-induced hypotension can reduce cerebral perfusion, potentially exacerbating ischemic injury in vulnerable aged brains. Some studies have also suggested that propofol may directly inhibit mitochondrial respiratory chain complexes or induce aberrant autophagy under certain conditions (61, 62). Therefore, the net effect of propofol on POCD risk in elderly patients likely depends on dosing, hemodynamic management, and individual susceptibility. Its recommendation as a uniformly neuroprotective strategy is premature; anesthetic selection should be individualized.

Beyond propofol, other intravenous adjuncts also show potential in modulating ferroptosis or related pathways. Dexmedetomidine (DEX), a highly selective α2-adrenergic receptor agonist with sedative, analgesic, and anxiolytic properties, reduces POCD incidence when used perioperatively (63). Its mechanisms are multifaceted: DEX attenuates hippocampal neuronal apoptosis, neuroinflammation, and DNA damage in aged mice (64), and reduces inflammation around the hippocampus and heart following cardiac surgery (64). In a lipopolysaccharide (LPS)-induced neuroinflammation model in aged mice, DEX significantly alleviated cognitive impairment and reduced elevated hippocampal levels of IL-6, ROS, ferritin light chain (FTL), and TfR1, indicating it corrects iron dyshomeostasis indirectly by inhibiting neuroinflammation and oxidative stress (65). Studies on esketamine have revealed its potential to intervene in preoperative sleep disturbance, a risk factor for POCD. Wen et al. found that esketamine reversed impaired hippocampal synaptic plasticity caused by sleep disturbance combined with surgery. It also inhibited microglial M1 polarization and inflammation while modulating the BDNF-TrkB signaling pathway, thereby ameliorating POCD and postoperative depressive symptoms in rats (66). Although this study did not directly investigate ferroptosis, microglial M1 polarization is closely linked to iron metabolism dysregulation, providing a foundation for future research (**Table 1**).

**Table 1:** Effects of different anesthetics on ferroptosis and related pathways.

Anesthetic	Type	Effect on ferroptosis	Mechanisms	Ref.
Sevoflurane	Inhalational	Promotes	Upregulates ACSL4, MIB2, PLCG1; Inhibits GPX4; Activates JNK/p53 pathway; Induces iron overload	(47, 48, 51, 53, 54)
Propofol	Intravenous	Inhibits (preclinically)/Risk of hemodynamic and mitochondrial toxicity	Activates Nrf2/GPX4, SIRT1 pathways; Antioxidant, anti-inflammatory, modulates eNOS/NO; may cause hypotension and mitochondrial complex inhibition	(58–60)
Dexmedetomidine	Sedative adjunct	Indirectly Inhibits	Anti-inflammatory, antioxidant, modulates iron metabolism proteins (FTL, TfR1)	(64, 65)
Esketamine	Intravenous	Potentially Inhibits	Modulates microglial polarization, BDNF-TrkB pathway	(66)

### 3.3 The multifactorial impact of surgical trauma

Surgical trauma is another core element triggering POCD, inducing a more complex and intense pathological response than anesthesia alone. The particularly high incidence of POCD following cardiac and major orthopedic surgery (e.g., hip arthroplasty) underscores the importance of surgical type. Surgical trauma intersects with ferroptosis mechanisms through several pathways. Firstly, it can induce metabolic reprogramming, polarizing microglia toward a pro-inflammatory state characterized by the release of IL-1β, IL-6, and TNF-α. It is now recognized that microglial responses in neurodegeneration and acute injury extend well beyond the simplified M1/M2 dichotomy; single-cell transcriptomics have revealed disease-associated microglial (DAM) signatures and neurotoxic subpopulations driven by damage-associated molecular patterns (DAMPs). Surgical trauma releases a multitude of DAMPs—including HMGB1, S100 proteins, and mitochondrial DNA—that activate Toll-like receptors (TLR4, TLR2) and the cGAS-STING pathway (67). IL-6 can directly impair synaptic plasticity (68) and, via STAT3 activation synergistically with bone morphogenetic proteins (BMPs), enhance hepcidin expression in astrocytes (69). Hepcidin, a master regulator of iron metabolism, binds to Fpn, inducing its internalization and degradation, thereby blocking neuronal iron release and leading to intracellular iron retention and surge (70). Thus, surgery-induced neuroinflammation can directly cause cerebral iron dysregulation via the IL-6/Hepcidin axis. Astrocytes, as central regulators of brain iron homeostasis, undergo reactive changes with age and injury, and can contribute to neuronal iron overload through the release of hepcidin, iron-containing exosomes, or ferritin. Emerging evidence also points to the infiltration of peripheral T lymphocytes (CD4+ and CD8+ T cells) across the disrupted BBB; these cells may secrete IFN-γ and further modulate microglial phenotypes and neuronal iron metabolism, although their role in POCD is only beginning to be explored. A study in rats undergoing splenectomy demonstrated that surgical trauma, but not anesthesia alone, caused cognitive impairment 1–3 days post-surgery. This was accompanied by significantly elevated iron content in brain tissue (especially hippocampus), aberrant expression of iron metabolism proteins (ferritin, TfR1, IRP2), and exacerbated oxidative stress (71), directly confirming the hypothesis that surgical trauma induces brain iron overload.

Secondly, the postoperative surge in peripheral inflammatory cytokines further compromises BBB integrity, increasing its permeability (72). This not only allows more peripheral inflammatory cytokines to enter the brain, exacerbating neuroinflammation, but, more critically, opens a channel for peripherally derived iron (e.g., hemoglobin iron from damaged red blood cells) to enter the brain parenchyma (72), directly supplying ample iron for ferroptosis.

**Figure 1:**
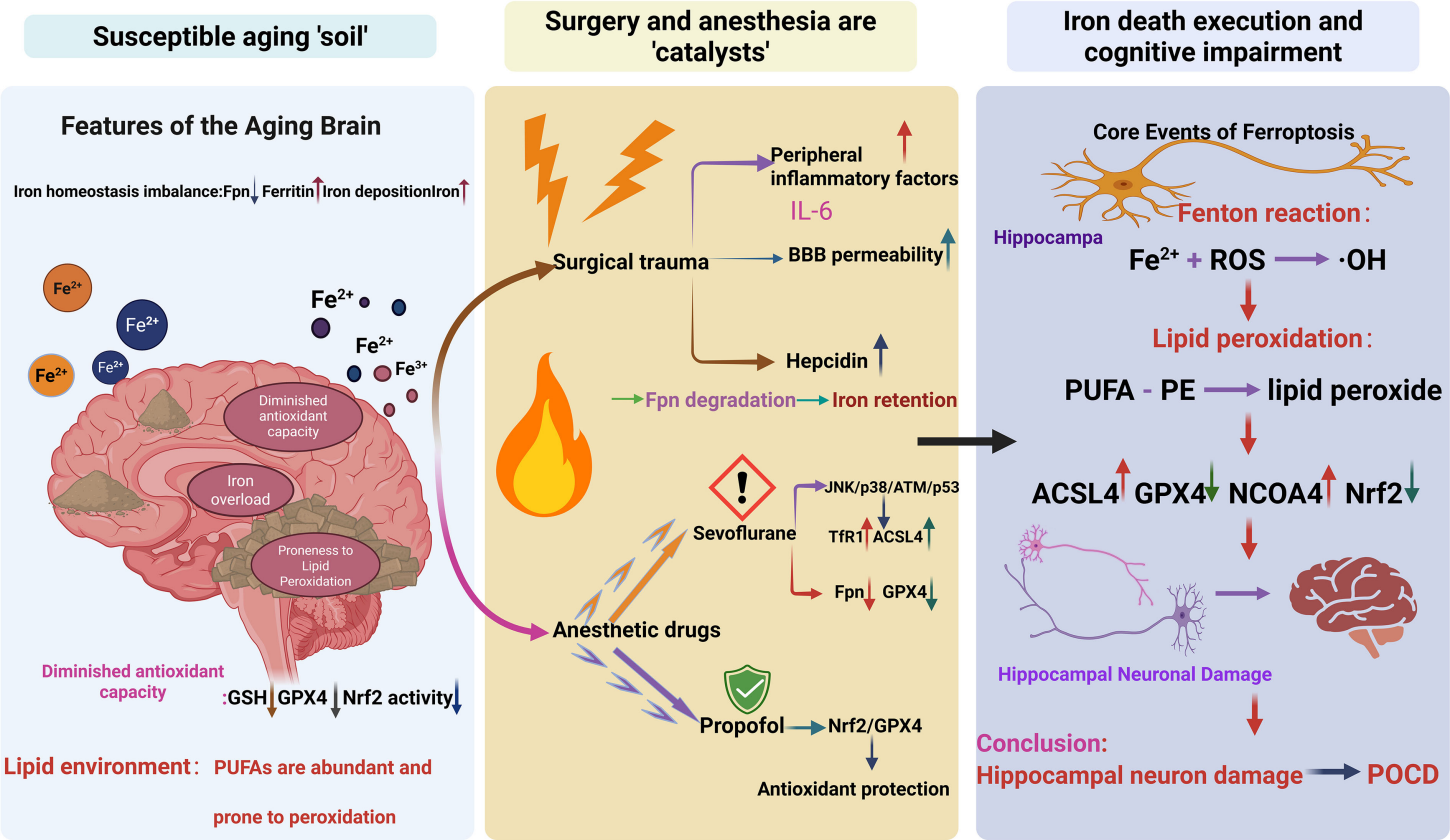
Schematic overview of ferroptosis as a core mechanism in POCD. Left panel: The aged brain exhibits disrupted iron homeostasis (decreased Fpn, increased iron deposition), diminished antioxidant capacity (decreased GPX4, decreased Nrf2 activity), and a lipid environment rich in polyunsaturated fatty acids (PUFAs), collectively establishing a ferroptosis-prone “soil”. Middle panel: Surgical trauma and anesthetic exposure act as “catalysts” by triggering neuroinflammation, disrupting the blood–brain barrier (BBB), inducing iron metabolic disturbances (e.g., the hepcidin/Fpn axis), and promoting oxidative stress, ultimately initiating ferroptosis. Right panel: Ferroptosis occurs in cognitive key regions such as the hippocampus, leading to neuronal death, synaptic dysfunction, and cognitive impairment, culminating in postoperative cognitive dysfunction (POCD). The causal sequence depicted here represents a testable hypothesis; ferroptosis may also occur as a downstream consequence of neuroinflammation and synaptic failure rather than as the initiating event. The relative timing and mechanistic primacy of these processes remain to be experimentally determined.

Furthermore, surgery is often accompanied by cerebral hypoxia, particularly during procedures like cardiopulmonary bypass (CPB). One study found that rats undergoing CPB surgery developed significant cerebral thrombosis and hypoxia in the hippocampal CA3 region, along with microglial activation, upregulated inflammatory cytokines, increased BBB permeability, and marked cognitive dysfunction (73). Cerebral hypoxia itself can regulate several iron metabolism genes (e.g., TfR1, DMT1) via the HIF-1α pathway, promoting cellular iron uptake while inhibiting mitochondrial function and increasing mitochondrial ROS production, thereby creating favorable conditions for the Fenton reaction and lipid peroxidation (**Figure 1**).

## 4 The molecular regulatory network of ferroptosis and its role in POCD

Against the backdrop of aging-related susceptibility, surgery and anesthesia converge through the aforementioned pathways onto a core molecular network, ultimately triggering ferroptosis. We first detail the well-established core machinery—GPX4, ACSL4, NCOA4, and Nrf2—and then discuss emerging regulators with varying degrees of evidence.

### 4.1 GPX4: the critical antioxidant barrier

GPX4, a selenoprotein, is the core enzyme responsible for reducing membrane lipid peroxides to non-toxic lipid alcohols, thereby terminating the lipid peroxidation chain reaction. Its activity is dependent on glutathione (GSH) as a substrate (74). Aging is accompanied by decreased GPX4 expression and activity in various tissues (75), rendering aged cells significantly more susceptible to ferroptosis. Perioperative stress may suppress the transcription of key protective genes like GPX4 by inducing aberrant DNA methylation patterns. Studies have shown that decreased Nrf2 activity in aged cardiomyocytes directly leads to reduced expression of antioxidant enzymes like GPX4 (76), and the use of GPX4 enzyme mimetics can alleviate stress-induced ferroptosis and maintain cognitive function in aged mice (77). Anesthetics like sevoflurane can block cystine uptake by inhibiting the glutamate transporter (System Xc-) on cell membranes, depleting intracellular cysteine (a substrate for GSH synthesis). This leads to insufficient GSH synthesis and ultimately GPX4 inactivation (78–80). MEF2C, a newly identified transcription factor, has been shown to directly bind and activate the Gpx4 gene promoter. In a POCD mouse model, MEF2C expression was downregulated, whereas overexpression of MEF2C significantly improved memory deficits and inhibited lipid peroxidation and iron accumulation. These protective effects were completely reversed by the GPX4 inhibitor RSL3 (81). This reveals the MEF2C/GPX4 axis as a key regulatory pathway essential for maintaining postoperative cognitive function.

### 4.2 ACSL4: the engine driving lipid peroxidation

Acyl-CoA synthetase long-chain family member 4 (ACSL4) is a crucial enzyme determining cellular sensitivity to ferroptosis. It preferentially catalyzes the conversion of long-chain polyunsaturated fatty acids (such as arachidonic acid C20:4 and adrenic acid C22:4) into their CoA derivatives, promoting their esterification into phospholipids, particularly phosphatidylethanolamine (PE), forming readily peroxidizable species like AA-PE (82, 83). These PUFA-PEs are ideal substrates for lipoxygenases (LOXs); their oxidation generates lipid peroxides (e.g., AA-OOH-PE), directly disrupting cell membrane structure. Elevated ACSL4 levels have been found in the substantia nigra of PD mouse models and PD patients, and inhibiting ACSL4 reduces lipid ROS and improves the PD phenotype (84). In the POCD field, research has confirmed that sevoflurane induces upregulation of ACSL4 in neurons, and silencing ACSL4 protects SH-SY5Y cells from sevoflurane-induced ferroptosis by activating the AMPK/mTOR signaling pathway (53). Furthermore, the traditional Chinese medicine formula Danggui Shaoyao San (DSS) has been shown to activate AMPK, disrupting its interaction with the transcription factor Sp1. This reduces Sp1 nuclear translocation and subsequently inhibits its transcriptional activation of the ACSL4 promoter, ultimately reducing neuronal ferroptosis in an AD mouse model (85). These findings indicate that targeting ACSL4 or its upstream regulatory signals is an effective strategy for intervening in ferroptosis.

### 4.3 NCOA4: the iron release switch

Ferritin serves as the primary intracellular iron storage depot. Nuclear receptor coactivator 4 (NCOA4)-mediated selective autophagy, termed ferritinophagy, is a critical pathway controlling iron mobilization and release (86). Under pathological conditions, excessive activation of NCOA4, binding to ferritin heavy chain 1 (FTH1) and delivering ferritin to autolysosomes for degradation, results in a massive release of free iron from storage depots into the labile iron pool (LIP). This free ferrous iron catalyzes ROS generation via the Fenton reaction, driving lipid peroxidation and ferroptosis. Studies have found that in a mouse model of type 2 diabetes mellitus (T2DM) with associated cognitive dysfunction, melatonin (MLT) inhibits excessive NCOA4-mediated ferritinophagy in brain tissue, thereby ameliorating learning and memory deficits (87). Notably, NCOA4-mediated ferritinophagy is not entirely detrimental. Ding et al. discovered that cyclosporine A (CsA) promotes the nucleocytoplasmic translocation of the RNA-binding protein HuR, which in turn upregulates NCOA4 expression, enhancing ferritinophagy. This moderate iron mobilization paradoxically inhibited microglial activation, reduced neuronal apoptosis, and improved cognitive function in mice (88). This finding reveals the complexity of NCOA4 regulation: moderate iron mobilization may represent a protective adaptation for cells under stress, while excessive activation leads to ferroptosis. This double-edged sword effect warrants further investigation.

### 4.4 Nrf2: the master conductor of antioxidant defense

Nuclear factor erythroid 2-related factor 2 (Nrf2) is the core transcription factor orchestrating cellular responses to oxidative and electrophilic stress, often considered the master regulator of the antioxidant defense system (89). Under physiological conditions, Nrf2 is sequestered in the cytoplasm bound to its inhibitor protein Keap1, leading to its ubiquitination and proteasomal degradation. Upon oxidative stress, Keap1 undergoes conformational changes or modifications, stabilizing Nrf2 and allowing its translocation into the nucleus. There, it forms heterodimers with small Maf proteins, binding to antioxidant response elements (ARE) in the promoter regions of target genes and initiating the transcription of a battery of protective genes (90). These target genes constitute a multi-layered defense against ferroptosis, including: (1) GPX4, which directly inhibits lipid peroxidation; (2) SLC7A11, the core component of System Xc- that provides the GSH substrate for GPX4; (3) Ferritin subunits (FTH1, FTL) and heme oxygenase-1 (HO-1) involved in iron sequestration and metabolism (91). Therefore, the level of Nrf2 activity directly determines the cellular “threshold” for ferroptosis.

**Figure 2:**
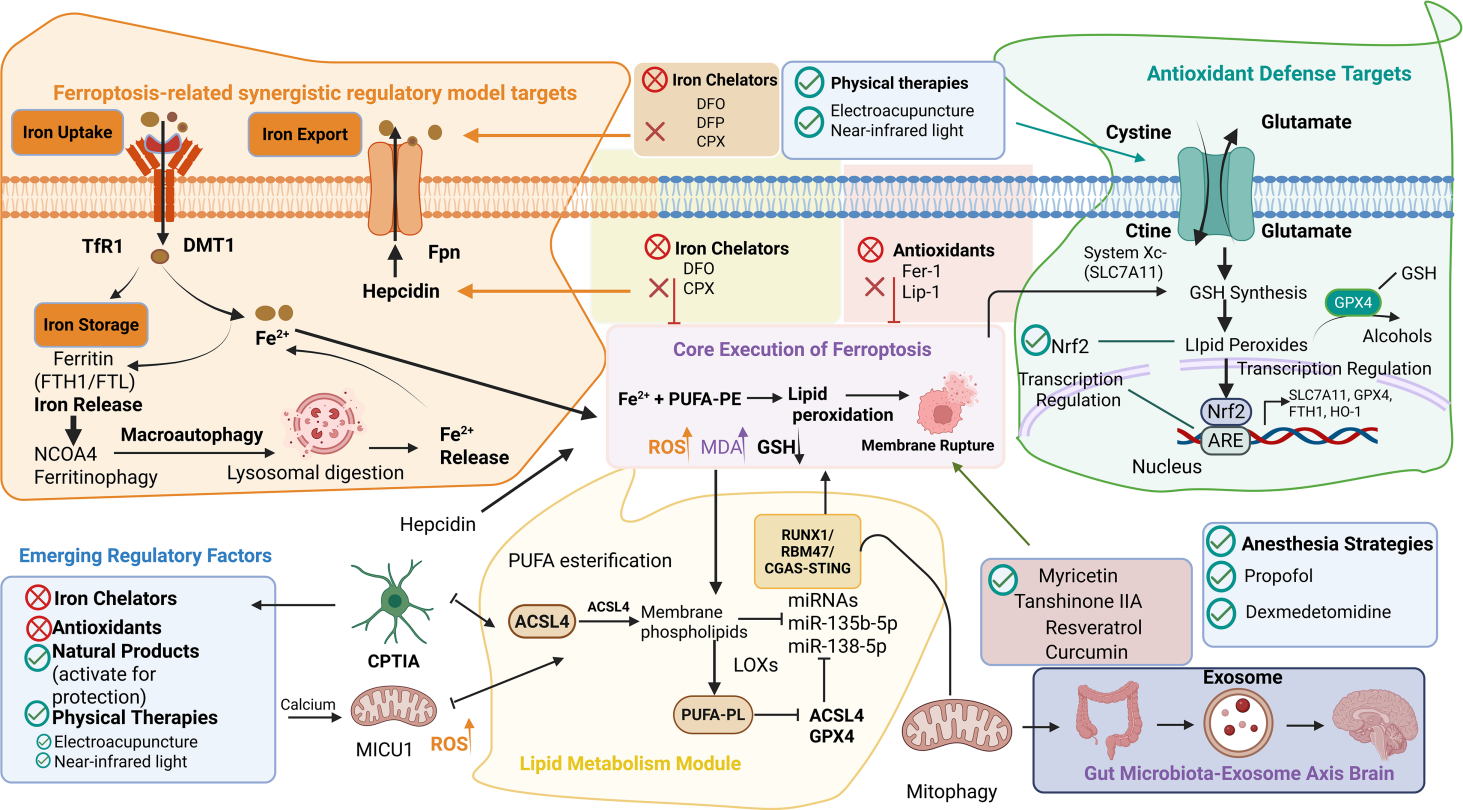
Molecular regulatory network of ferroptosis and therapeutic targets. Ferroptosis is driven by iron-dependent lethal accumulation of lipid peroxides. Key regulatory modules include: ① Iron metabolism (uptake via TfR1/DMT1, release of Fe²^+^ via NCOA4-mediated ferritinophagy, and efflux via Fpn); ② Antioxidant defense (System Xc⁻/GSH/GPX4 axis and Nrf2-mediated transcriptional regulation); ③ Lipid peroxidation (ACSL4-facilitated esterification of PUFAs into membrane phospholipids and oxidation by LOXs). Core pathways (GPX4, ACSL4, NCOA4, Nrf2) with robust evidence are distinguished from emerging regulators (MD2/hepcidin, CPT1A, MICU1, RUNX1/RBM47/cGAS-STING, miRNAs, mitophagy, gut microbiota-exosome axis) that require further validation. Therapeutic interventions (iron chelators, lipophilic antioxidants, natural products, physical therapies) are indicated; most remain at the preclinical stage. Solid arrows denote pathways supported by multiple independent studies; dashed arrows denote more preliminary or context-dependent associations. Red denotes promotion or upregulation; green denotes inhibition or downregulation.

In the pathological context of POCD, the Nrf2 pathway is significantly inhibited: sevoflurane anesthesia leads to a marked decrease in both mRNA and protein levels of Nrf2 in the hippocampus of aged mice, with concomitant reductions in its downstream targets HO-1, GPX4, and SLC7A11 (52). Myricetin exerts its effects precisely by reversing sevoflurane-induced HDAC2 upregulation and Nrf2 deacetylation, thereby restoring Nrf2 transcriptional activity, activating downstream protective pathways, and inhibiting ferroptosis (52). Moreover, numerous natural products and drugs with neuroprotective properties have been shown to exert their effects via Nrf2 pathway activation. Tanshinone IIA alleviates hippocampal inflammation and ferroptosis in aged POCD rats by activating the Nrf2/SLC7A11/GPX4 axis (92). Avenanthramide-C (AVC) inhibits apoptosis, neuroinflammation, and ferroptosis induced by repeated propofol anesthesia in the hippocampus of aged rats by activating the Nrf2/ARE pathway (93). Electroacupuncture (EA) treatment has also been found to inhibit ferroptosis and improve cognitive function in POCD mice by modulating the GRX1/GSK-3β/Nrf2 pathway (94). The short-chain fatty acids sodium acetate (NaA) and sodium butyrate (NaB) significantly activate the downstream antioxidant enzymes SOD and GPx via Nrf2 while improving cognitive function in aged POCD rats (95). Echinatin similarly attenuates sevoflurane-induced neurotoxicity and ferroptosis by activating Nrf2 signaling (96). These studies collectively establish Nrf2 as a central regulatory hub in ferroptosis and a common target for diverse intervention strategies (**Figure 2**).

### 4.5 Emerging regulatory factors and signaling axes

Beyond the core machinery described above, several additional modulators have been linked to ferroptosis in POCD models. Their mechanistic roles vary from well-supported to highly speculative; we briefly discuss those with converging evidence and then flag more preliminary findings.

Myeloid differentiation protein 2 (MD2) is an accessory protein for Toll-like receptor 4 (TLR4), primarily responding to exogenous pathogen signals. In an aged mouse model of POCD following splenectomy, MD2 transgenic mice exhibited more severe cognitive impairment and hippocampal ferroptosis, accompanied by upregulated expression of MD2, hepcidin, and TFR. Inhibiting MD2 significantly improved symptoms, suggesting MD2 regulates neuronal ferroptosis by promoting hepcidin expression, positioning it as a novel therapeutic target for POCD (97). This finding directly links an innate immune receptor with a core hormone of iron metabolism.

Carnitine palmitoyltransferase 1a (CPT1A) is the rate-limiting enzyme in mitochondrial fatty acid oxidation. In a co-culture system of SVG P12 astrocytes and hippocampal neurons, CPT1A overexpression enhanced astrocytic mitochondrial function, which in turn improved neuronal synaptic integrity, reduced oxidative stress, maintained iron homeostasis, and inhibited ferroptosis. Conversely, reduced CPT1A levels exacerbated neuronal injury (98). This indicates that the lipid metabolic state of astrocytes directly influences the ferroptosis sensitivity of neurons, providing novel insights into intercellular crosstalk mechanisms in POCD. Mitochondrial calcium uptake 1 (MICU1) is a critical protein for maintaining mitochondrial calcium homeostasis. In a POCD rat model, MICU1 expression was decreased, whereas overexpression of MICU1 inhibited hippocampal neuronal ferroptosis and alleviated brain tissue damage by maintaining calcium homeostasis and reducing ROS production (99). This study reveals a potential link between mitochondrial calcium signaling and ferroptosis.

Transcriptomic analysis identified significant upregulation of the RNA-binding protein Rbm47 in the hippocampus of POCD mice. Mechanistic studies revealed that the transcription factor RUNX1 directly binds to the Rbm47 promoter, promoting its expression. RBM47, in turn, binds to and stabilizes cGAS mRNA via its RRM2 domain, thereby activating the cGAS-STING pathway, which mediates neuroinflammation and neuronal ferroptosis. Crucially, the triggers that activate cGAS in the sterile surgical/aging microenvironment are likely mitochondrial DNA (mtDNA) released into the cytosol as a result of oxidative damage and impaired mitophagy, as well as genomic instability in aged neurons. This mtDNA is sensed by cGAS, driving STING-dependent IRF3/NF-κB activation and linking mitochondrial distress to innate immunity and ferroptosis. Targeting RUNX1 or RBM47 significantly alleviated POCD (100). This signaling axis integrates transcriptional regulation, RNA stability, and inflammatory-ferroptotic cascades, presenting novel intervention targets for POCD.

MicroRNAs (miRNAs) play significant roles in ferroptosis regulation. Besides the aforementioned miR-135b-5p (56), morin has been found to downregulate miR-138-5p, thereby relieving its inhibition of SIRT1. This activates the SIRT1/p53 pathway, upregulates SLC7A11 and GPX4, inhibits hippocampal ferroptosis, and improves POCD (101). This suggests that targeting miRNAs could be an effective strategy for modulating ferroptosis.

In a neurotoxicity model induced by hypoxia combined with propofol (HCWP), excessive mitophagy was identified as an upstream event of ferroptosis. HCWP triggered ferroptosis by inducing decreased mitochondrial membrane potential, ROS burst, and mitochondrial fragmentation. The mitophagy inhibitor Mdivi-1 effectively blocked this process (102). Although this observation is intriguing, it rests on a single *in vitro* study and requires independent replication *in vivo* before excessive mitophagy can be considered a validated driver of ferroptosis in POCD.

Finally, in a rat model of POCD following colorectal cancer surgery, researchers observed significant gut microbiota dysbiosis. Exosome-like nanoparticles derived from the gut microbiota were found to be taken up by hippocampal neurons, triggering ferroptosis markers (upregulation of ATG5, COX2; downregulation of GPX4, FTH1) and leading to cognitive decline (103). This provocative finding provides preliminary evidence for a gut-brain axis mechanism in POCD, but its generalizability and the exact molecular cargo of the exosomes remain to be defined.

### 4.6 Crosstalk between cell death modalities

Ferroptosis does not occur in isolation; it engages in extensive crosstalk with apoptosis, autophagy, necroptosis, and other cell death pathways, collectively determining neuronal fate. p53, the classic tumor suppressor, plays a bidirectional regulatory role between ferroptosis and apoptosis: it can promote ferroptosis by inhibiting SLC7A11 transcription while also directly inducing the expression of pro-apoptotic genes (104). The intersection of autophagy and ferroptosis is particularly prominent. NCOA4-mediated ferritinophagy acts as an upstream event for ferroptosis (86), and excessive autophagy can further trigger ferroptosis by degrading anti-ferroptotic proteins like GPX4. Additionally, oxidative stress can activate the NLRP3 inflammasome, inducing pyroptosis, which synergistically amplifies neuroinflammation alongside ferroptosis (42). Understanding this crosstalk is crucial for designing combination therapeutic strategies; for instance, simultaneously blocking ferroptosis and apoptosis might more effectively protect neurons. This also provides a theoretical basis for future multi-target drug combinations (**Table 2**).

**Table 2:** The molecular regulatory network of ferroptosis and its role in POCD.

Molecule/regulatory axis	Functional role	Mechanisms in POCD	Intervention potential	Ref.
GPX4	Antioxidant barrier	Aging or anesthetics (e.g., sevoflurane) cause GPX4 inactivation/degradation, leading to lipid peroxide accumulation and ferroptosis	GPX4 activators, MEF2C overexpression	(52, 74–81)
ACSL4	Drives PUFA incorporation into Pls	Sevoflurane upregulates ACSL4; silencing ACSL4 protects neurons via AMPK/mTOR pathway	ACSL4 inhibitors, AMPK activators	(53, 82–85)
NCOA4	Mediates ferritinophagy	Overactivation leads to iron release and exacerbated Fenton reaction; moderate activation may be protective	Modulating ferritinophagy balance	(86–88)
Nrf2	Master antioxidant conductor	Sevoflurane inhibits Nrf2 activity; agents like myricetin, Tanshinone IIA activate the Nrf2 pathway	Nrf2 agonists (natural products, electroacupuncture)	(52, 89–93, 95, 96, 105)
MD2/Hepcidin axis	Iron metabolism & immune response	MD2 promotes hepcidin expression, leading to iron retention	MD2 inhibitors	(97)
RUNX1/RBM47/cGAS-STING	Transcriptional & inflammatory	RUNX1 activates RBM47, stabilizing cGAS mRNA and activating the STING pathway; mitochondrial DNA (mtDNA) released due to oxidative damage acts as a key DAMP triggering cGAS in the sterile surgical microenvironment, inducing ferroptosis	Targeting RUNX1/RBM47	(100)
miR-135b-5p/miR-138-5p	Epigenetic regulation	miR-135b-5p regulates JAK2-STAT3-Hepcidin; miR-138-5p targets SIRT1	miRNA mimics or inhibitors	(56, 101)
Mitophagy	Mitochondrial quality control	Excessive mitophagy (e.g., in HCWP model) induces ferroptosis (limited evidence)	Autophagy inhibitors (e.g., Mdivi-1)	(102)
Gut microbiota-exosome axis	Systemic regulation	Dysbiosis releases exosomes, triggering hippocampal ferroptosis (preliminary)	Probiotics, SCFAs, fecal microbiota transplant	(103)

### 4.7 Limitations and alternative hypotheses: is ferroptosis a cause or a consequence?

A critical assessment of the literature reveals that most studies supporting a ferroptosis-centric model of POCD have demonstrated correlations between elevated ferroptosis markers and cognitive impairment, or that pharmacological inhibitors of ferroptosis improve outcomes. Such evidence, while suggestive, does not establish causality. An alternative hypothesis must be seriously entertained: that ferroptosis is a late-stage, terminal event occurring in neurons already rendered moribund by neuroinflammation and excitotoxicity. In this scenario, ferroptosis would be a consequence rather than a primary driver, and its inhibition might offer little functional benefit once upstream inflammatory damage has been initiated. If ferroptosis is indeed a late event, its value as a therapeutic target would be diminished, as intervention windows would be very narrow.

Distinguishing between these possibilities requires experiments that temporally resolve the sequence of events. For example, neuron-specific conditional knockout of Gpx4 or Acsl4 could be used to test whether blocking ferroptosis at the genetic level prevents POCD independently of inflammation. High-resolution time-course studies measuring synaptic function, cytokine levels, and lipid peroxidation markers in the hours to days following surgery could clarify whether ferroptotic changes precede or follow synaptic failure. Additionally, novel fluorescent probes that detect lipid peroxidation in real-time within specific cell types could establish the exact cellular and temporal origin of ferroptosis. Only when such evidence is available can ferroptosis be confidently designated as a core mechanism, rather than a terminal bystander, in POCD.

## 5 Therapeutic prospects and future directions

Based on the understanding of ferroptosis’s core role in POCD pathogenesis, intervention strategies targeting different nodes of the ferroptosis pathway hold considerable therapeutic promise. However, nearly all of these strategies are at the preclinical stage, and their translation to clinical practice faces significant hurdles.

### 5.1 Targeting iron metabolism: iron chelators

Iron chelators, which bind and sequester excess intracellular free iron, thereby cutting off the substrate for the Fenton reaction at its source, represent a classic strategy for directly inhibiting ferroptosis. Deferoxamine (DFO), a first-generation iron chelator, has demonstrated neuroprotective effects in several POCD-related models. Li et al. found that aged mice undergoing abdominal surgery still exhibited hippocampal ferroptosis 14 days post-operation. DFO preconditioning effectively ameliorated the downregulation of Fpn, overexpression of hepcidin and DMT1, reduced inflammatory cytokine levels, and controlled memory deficits (106, 107). In a lipopolysaccharide (LPS)-induced neuroinflammation model, DFO similarly improved memory deficits and reduced hippocampal iron accumulation, microglial activation, and inflammatory cytokine release (108, 109). Notably, as early as 1993, a small clinical study in AD patients observed that DFO could slow the decline in daily living abilities (110), although its inability to cross the BBB limits its application. Second-generation iron chelators capable of crossing the BBB, such as deferiprone, have shown more promising effects in AD mouse models (111) and reduced hippocampal Aβ and p-tau phosphorylation in a high-fat diet rabbit model (112). Other iron chelators like ciclopirox olamine (CPX) and deferasirox (DFX) have also demonstrated potential in inhibiting ferroptosis (113). Therefore, the development of novel iron chelators with improved safety profiles and enhanced BBB permeability is an important future direction.

### 5.2 Targeting lipid peroxidation: lipophilic antioxidants

Ferrostatin-1 (Fer-1) and Liproxstatin-1 (Lip-1) are potent lipophilic antioxidants that directly interrupt the lipid peroxidation chain reaction by trapping lipid peroxyl radicals or inhibiting lipoxygenase activity. Intrathecal injection of Fer-1 attenuated bupivacaine-induced spinal neurotoxicity (78) and reduced lesion size while improving long-term outcomes in TBI models (114). Lip-1 has been shown to be protective in various ischemia-reperfusion injury models (e.g., liver, heart, kidney, lung transplantation) (115–117), with mechanisms potentially involving upregulation of SLC7A11 and reduction of inflammatory cytokine release. Given that surgical trauma is often accompanied by local tissue ischemia-reperfusion, the potential application of such agents in POCD prevention and treatment warrants further investigation.

### 5.3 Targeting upstream regulatory pathways: natural products and physical therapies

Numerous natural products, acting on upstream regulators like Nrf2 and SIRT1, exhibit multi-target, synergistic protective effects. Recent reviews have emphasized that many plant-derived compounds act as geroprotectors and ferroptosis modulators by targeting aging hallmarks, AMPK-linked metabolic pathways, and antioxidant defense systems (118–120). In addition to the aforementioned myricetin (52), Tanshinone IIA (92), AVC (93), and echinatin (96), resveratrol has been shown to activate the Nrf2/HO-1 pathway (121)and SIRT1 (122, 123), and promote microglial polarization toward an anti-inflammatory M2 phenotype by modulating the CX3CL1/CX3CR1 axis (123). Curcumin enhances SOD activity, increases BDNF expression, and prevents cholinergic dysfunction via the Nrf2-HO-1 pathway (124). Fucoxanthin not only alleviates neuroinflammation through the pAkt and ERK pathways but also enhances antioxidant enzyme activity (125). Morin was found to inhibit ferroptosis by downregulating miR-138-5p, thereby activating the SIRT1/p53 pathway (101). These findings indicate that the treasure trove of traditional Chinese medicine contains abundant candidate drugs for POCD treatment. It is critical to note, however, that all evidence for natural products in the context of POCD is currently preclinical; none of these compounds has been validated in randomized controlled trials, and therefore they should be regarded as promising candidates rather than established therapies.

Physical therapies such as electroacupuncture and near-infrared light also demonstrate unique application value. Electroacupuncture (EA) at the Baihui acupoint (GV20) has been shown to inhibit hippocampal ferroptosis and improve cognitive function in POCD mice by modulating the GRX1/GSK-3β/Nrf2 pathway (94) or regulating the TFR1-DMT1-FPN pathway (126). Near-infrared (NIR) light has been found to reverse glutamate-induced neuronal ferroptosis by targeting the Lipocalin-2 (Lcn2) pathway and improve cognitive function in sevoflurane-induced POCD mice (127). As a non-invasive physical therapy with a high safety profile, NIR holds significant potential for clinical translation.

Regarding anesthetic strategies, clinical studies on the relationship between anesthesia depth and POCD risk remain controversial. One study in young to middle-aged healthy individuals found that the degree of EEG suppression under general anesthesia was not associated with cognitive decline (128). A meta-analysis also suggested no significant difference in MMSE scores related to anesthesia depth (129). However, studies focusing on elderly patients aged over 65 indicate that the incidence of POCD is lower with light general anesthesia (BIS 60-80) compared to deep anesthesia (BIS 40-60) (130). These conflicting results may be attributed to differences in study populations, surgical types, and cognitive assessment methods. Regarding anesthetic drug selection, while propofol has shown preclinical antioxidant advantages, its clinical superiority over sevoflurane in preventing POCD remains to be definitively proven, and the choice must balance pharmacological properties against hemodynamic stability and the risk of propofol-related toxicity. The incorporation of adjuncts like dexmedetomidine appears beneficial, but current evidence is insufficient to recommend a universal anesthetic regimen for elderly patients; individualized approaches based on patient frailty and comorbidities are prudent.

### 5.4 Emergence of new targets and systemic regulation

As our understanding of the ferroptosis regulatory network deepens, a series of new targets have been revealed, offering further possibilities for POCD treatment. For example, MD2 (95, 97), CPT1A (98), MICU1 (99), the RUNX1/RBM47 axis (100), as well as miR-138-5p (101) and miR-135b-5p (56), have all been implicated in POCD-related ferroptosis, providing new candidate molecules for drug development. Furthermore, the discoveries regarding mitophagy (102) and the gut microbiota-exosome axis (103) expand our understanding of the regulatory dimensions of ferroptosis. The effects of the short-chain fatty acids sodium acetate (NaA) and sodium butyrate (NaB) in mitigating ferroptosis by inhibiting the cGAS-STING pathway have been confirmed in multiple studies (95), further reinforcing the potential of the “gut-brain axis” in POCD prevention and treatment.

In recent years, advances in nanotechnology have provided novel strategies for POCD management. Researchers have synthesized mannose-modified superparamagnetic iron oxide nanoparticles (mSPIONs) with a small particle size (approx. 11 nm), enabling effective BBB penetration. In a mouse model of tibial fracture surgery, mSPIONs significantly attenuated neuroinflammation and improved postoperative cognitive function by scavenging excess ROS and inhibiting the NF-κB pathway (131). This nanozyme, combining iron-chelating and antioxidant functions, demonstrates promising potential for clinical translation.

### 5.5 Translational challenges: biomarkers, imaging, and clinical trial design

A major barrier to translating ferroptosis-targeted therapies for POCD is the lack of validated biomarkers that can identify ferroptosis in the living human brain and distinguish it from other forms of oxidative injury. While traditional markers such as plasma MDA, 4-HNE, and reduced GSH indicate general oxidative stress, more specific indicators—such as the lipid peroxidation product 15-HpETE-PE, GPX4 activity in cerebrospinal fluid, or the ratio of labile iron to ferritin—may better reflect ferroptosis and should be evaluated. Non-invasive MRI techniques, including quantitative susceptibility mapping (QSM) and T2* mapping, can estimate regional brain iron content and have been correlated with cognitive decline in neurodegenerative diseases. Pilot studies suggest that susceptibility changes in the basal forebrain may be associated with postoperative delirium (129). However, no prognostic biomarker for POCD has been prospectively validated. Future clinical studies must integrate multimodal biomarkers—imaging, CSF, and blood—to construct risk prediction models and to monitor target engagement. Furthermore, clinical trial design for ferroptosis inhibitors must carefully select patients with evidence of pre-existing cerebral iron dyshomeostasis, define optimal intervention timing, and employ sensitive cognitive and functional endpoints over an adequate follow-up period.

In summary, based on a deep understanding of the core mechanisms of ferroptosis, future POCD prevention and treatment strategies should shift from single-target interventions toward multi-dimensional, integrated systemic regulation. This involves combining approaches that target iron metabolism, lipid peroxidation, upstream signaling networks, and the gut-brain axis, leveraging novel delivery platforms like nanotechnology. Ultimately, this aims to achieve a breakthrough from basic research to precise clinical translation, providing safer and more effective personalized diagnostic and therapeutic paradigms for high-risk elderly patients (**Table 3**).

**Table 3:** Intervention strategies targeting ferroptosis and representative agents.

Intervention strategy	Representative agents/methods	Mechanism of action	Research progress	Ref.
Iron Chelators	Deferoxamine (DFO), Deferiprone, Deferasirox (DFX)	Bind free iron, blocking the Fenton reaction	DFO improves cognition in POCD models; Deferiprone crosses BBB	(106–113)
Lipophilic Antioxidants	Ferrostatin-1, Liproxstatin-1	Trap lipid radicals, inhibit lipid peroxidation	Effective in ischemia-reperfusion and neurotoxicity models	(78, 114–117)
Natural Products	Myricetin, Tanshinone IIA, Resveratrol, Curcumin, Fucoxanthin, Morin	Activate Nrf2/SIRT1 pathways, inhibit ferroptosis	Multi-target, low toxicity, effective in aged POCD models; no clinical trials	(52, 92, 93, 95, 96, 105, 121–125)
Physical Therapies	Electroacupuncture (EA), Near-infrared light (NIR)	Modulate iron metabolism pathways (e.g., TFR1-DMT1-FPN), inhibit Lcn2 pathway	Improve cognitive function, high safety profile	(105, 126, 127)
Nanomaterials	mSPIONs (Mannose-coated SPION nanozyme)	Penetrate BBB, scavenge ROS, inhibit NF-κB pathway	Neuroprotective in fracture model	(131)
Anesthetic Optimization	Propofol (vs. sevoflurane), Dexmedetomidine	Activate SIRT1/Nrf2/GPX4 pathway, antioxidant, anti-inflammatory	Mixed clinical evidence; no universal recommendation	(60, 63–65, 130, 132, 133)

## 6 Conclusions and future perspectives

The hypothesis that ferroptosis functions as a central pathogenic mechanism in POCD is compelling but requires more rigorous validation before it can be considered an established framework. Aging-associated iron accumulation, impaired antioxidant defenses, and PUFA-rich neuronal membranes create a permissive environment for ferroptosis, while surgical trauma and certain anesthetics provide the precipitating triggers. The protective effects of iron chelators, lipophilic antioxidants, and Nrf2-activating natural products in preclinical POCD models lend support to this concept. However, these observations remain largely correlative, and a critical unresolved question persists: does ferroptosis act as a proximal executor of synaptic damage and neuronal death, or is it merely a terminal removal mechanism for neurons already rendered non-functional by neuroinflammation and metabolic stress?

Addressing this central question requires several lines of investigation. First, temporally resolved studies are needed to determine whether lipid peroxidation and GPX4 inactivation precede or follow synaptic failure and microglial activation in the hours to days after surgery. Second, neuron-specific conditional knockout models (e.g., Gpx4 or Acsl4 deletion restricted to hippocampal neurons) should be employed to test whether blocking ferroptosis at the genetic level prevents POCD independently of anti-inflammatory effects. Third, the field must clarify which cell types—neurons, astrocytes, microglia, or oligodendrocytes—are the primary sites of ferroptotic damage, and whether intercellular propagation of lipid peroxides contributes to disease progression.

Equally important are the translational challenges. Reliable biomarkers that can distinguish ferroptosis from other forms of oxidative injury in living patients are currently lacking. While QSM and T2 MRI can quantify regional brain iron content, and candidate lipid peroxidation products such as 15-HpETE-PE can be measured in cerebrospinal fluid, none has been prospectively validated for POCD risk stratification. Future clinical studies should integrate multimodal biomarkers—neuroimaging, CSF analysis, and blood-based assays—to construct predictive models and to monitor target engagement during therapeutic trials. Furthermore, key questions regarding therapeutic intervention remain unanswered: Is there a critical temporal window beyond which anti-ferroptotic therapy becomes futile? Would combining ferroptosis inhibitors with anti-inflammatory agents yield synergistic benefits, or merely add toxicity? Can BBB-penetrant iron chelators or GPX4 activators be safely administered to frail elderly surgical patients without compromising systemic iron homeostasis or wound healing? Answers to these questions will determine whether ferroptosis-targeted therapies can progress from promising preclinical candidates to clinically meaningful interventions.

Ultimately, the ferroptosis-centric model of POCD represents an exciting but still developing framework. The uncertainties outlined above should not be viewed as weaknesses of the hypothesis, but rather as guideposts for the next generation of mechanistic and translational research. It is only through carefully designed studies that directly test causality, establish temporal dynamics, and bridge the gap between animal models and human disease that the true role of ferroptosis in POCD will be elucidated—and its therapeutic potential realized.
