# Supplementary material for: Ferroptosis: an emerging key mechanism linking aging, surgical and anesthetic exposure to postoperative cognitive dysfunction
Source: Front Immunol. 2026 May 22;17:1836516. doi: 10.3389/fimmu.2026.1836516 (PMC13237643; doi:10.3389/fimmu.2026.1836516)
Supplement: Supplementary file 1 [file Table1.docx]

Supplementary Table 1: Summary of Key POCD/Ferroptosis Studies

| Model (species/cell) | Intervention/Target | Pathway/Marker Assessed | Main Outcome | Reference |
| --- | --- | --- | --- | --- |
| Aged rat splenectomy | DFO pretreatment | Iron, Fpn, hepcidin, inflammation | Reduced iron overload & memory deficit | (105). |
| Neonatal mouse sevoflurane | Iron chelator, p53 inhibitor | ATM/p53, TfR1, Fpn, lipid peroxidation | Reduced neuron death & cognitive impairment | (51) |
| SH-SY5Y sevoflurane | ACSL4 siRNA | AMPK/mTOR, GPX4, ROS | Protected from ferroptosis | (53) |
| Aged mouse splenectomy | Propofol vs. isoflurane | SIRT1/Nrf2/GPX4, ACSL4 | Propofol inhibited ferroptosis & improved cognition | (60) |
| Aged mouse POCD | Myricetin | HDAC2/Nrf2/HO-1 | Inhibited ferroptosis & mitochondrial damage | (52) |
| Mouse tibial fracture | mSPIONs | ROS, NF-κB | Attenuated neuroinflammation & cognitive decline | (130) |
| POCD mouse model | MEF2C overexpression | Gpx4, lipid peroxidation | Improved memory, reversed by RSL3 | (81) |
| POCD mouse model | RUNX1/RBM47 knockdown | cGAS-STING, MEF2C | Alleviated neuroinflammation & ferroptosis | (100) |
| Aged rat POCD | Tanshinone IIA | Nrf2/SLC7A11/GPX4 | Reduced hippocampal inflammation & ferroptosis | (92) |
| Aged mouse LPS | Dexmedetomidine | IL-6, ROS, FTL, TfR1 | Corrected iron dyshomeostasis | (65) |
